# Empowering patients in decision‐making in radiation oncology – can we do better?

**DOI:** 10.1002/1878-0261.12675

**Published:** 2020-04-13

**Authors:** Michelle Leech, Matthew S. Katz, Joanna Kazmierska, Julie McCrossin, Sandra Turner

**Affiliations:** ^1^ Applied Radiation Therapy Trinity Research Group Discipline of Radiation Therapy School of Medicine Trinity College Dublin Ireland; ^2^ Department of Radiation Medicine Lowell General Hospital MA USA; ^3^ Greater Poland Cancer Centre Poznan Poland; ^4^ Voluntary Patron, Targeting Cancer, BeyondFive TROG Cancer Research Sydney NSW Australia; ^5^ Radiation Oncology Department Westmead Hospital Sydney NSW Australia

**Keywords:** patient empowerment, radiation therapy, shared decision‐making

## Abstract

The decision as to whether or not a patient should receive radiation therapy as part of their cancer treatment is based on evidence‐based practice and on recommended international consensus treatment guidelines. However, the merit of involving the patients' individual preferences and values in the treatment decision is frequently overlooked. Here, we review the current literature pertaining to shared decision‐making (SDM) in the field of radiation oncology, including discussion of the patient's perception of radiation therapy as a treatment option and patient involvement in clinical trials. The merit of decision aids during the SDM process in radiation oncology is considered, as are patient preferences for active or passive involvement in decisions about their treatment. Clarity of terminology, a better understanding of effective strategies and increased resources will be needed to ensure SDM in radiation oncology becomes a reality.

## Graphical abstract

Radiation therapy is a form of cancer treatment recommended for approximately half of all cancer patients. The patient as a decision‐maker in radiation therapy is frequently overlooked. This review explores the patient perception of radiation therapy as a treatment option and the evidence for using aids in assisting patients to make a decision about their cancer care.

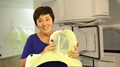

**Abbreviations**
EBRT: external beam radiation therapy
IPDAS: International Patient Decision Aids Standards
NICE: National Institute of Clinical Excellence
NSCLC: non‐small‐cell lung cancer
PRO: patient‐reported outcome
PROM: patient‐reported outcome measure
RP: radical prostatectomy
SABR: stereotactic ablative radiation therapy
SDM: shared decision‐making
SMOG: Simple Measure of Gobbledygook
TROG: Trans‐Tasman Radiation Oncology Group
VCM: values clarification method

## 1 Introduction

### 1.1 The patient as a key decision‐maker

Advances in molecular oncology have revolutionised the ability of healthcare professionals to select specific cancer treatments for patients that are most likely to respond, and have aided in treatment guideline development. Personalised medicine is an evolving approach to patient care in which each individual patient's characteristics guide clinical decisions, with a view to providing the optimal treatment to the patient at the right time (Jackson and Chester, 2015). Recent examples include the addition of temozolomide to radiation therapy for the management of glioblastoma multiforme depending on methyl guanine methyl transferase methylation status (Stupp *et al.*, 2002), the potential for dose de‐escalation of radiation therapy for human papillomavirus‐positive oropharyngeal carcinomas (Wirth *et al.*, 2019) and the definition of five molecular subtypes of breast cancer, based on the St. Gallen consensus criteria (Balic *et al.*, 2019).

Patient empowerment, patient engagement, patient advocacy and patient involvement are terms that are now frequently used in the move towards personalisation of health care. Although these phrases are used interchangeably in the radiation oncology‐specific literature, each describes a distinct concept.

Patient empowerment represents the patient's increased desire and ability to take part in care. Patient empowerment is often seen as a function of patient confidence in their status within healthcare systems. Patient engagement can be defined as ‘the desire and capability to actively choose to participate in care in a way uniquely appropriate to the individual in cooperation with a healthcare provider or institution for the purposes of maximising outcomes or experiences of care’ (Higgins *et al.*, 2017).

Patient involvement has been described by Graffigna and Vegni (2017) as the bilateral context of the doctor‐clinician consultation in shared decision‐making (SDM). Patient advocacy promotes autonomy of patients through patient‐centred decision‐making and ensuring appropriate availability and use of services and thereby improving the quality of services (Blankenship and Duffy, 2015). Clearly, these concepts overlap and are partly interdependent, a situation reflected in the available literature. Stronger patient engagement may lead to increased empowerment or involvement and equally the processes associated with empowering and involving patients may contribute to a state of increased patient engagement (Higgins *et al.*, 2017).

Clinicians frequently overlook the inclusion of patient preferences and value systems in treatment decisions, most likely due to their lack of recognition of the importance of empowering patients to make decisions regarding their own treatment trajectories. In order to empower patients to take ownership of their cancer management plan, they must be part of the treatment decision‐making process. Such ‘shared decision‐making’ involves the patient and clinician sharing information and working towards consensus about the most suitable option on how to proceed (Barry, 2010). Other key components of SDM are as follows: respect of patients' preferences; provision of timely, balanced and individualised information; and consideration of family and cultural parameters that may influence (Feuz, 2014).

**Fig. 1:**
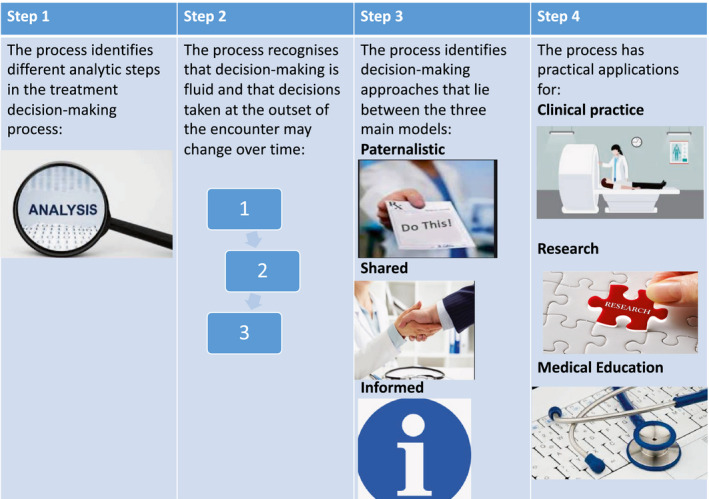
Schematic representation of SDM framework, as defined by Charles *et al.* (1999). Decision‐making is a fluid process and can change over time. The framework's model lies between paternalistic, shared and informed and impacts on clinical practice, research and education of health professionals.

National Institute for Health and Care Excellence (2004) practice guidelines recognise patients as key players in the decision‐making regarding their own care. SDM has also been incorporated into the European Cancer Patients Bill of Rights (Hojgaard *et al*., 2016), the Patient Protection and Affordable Care Act (Manchikanti *et al.*, 2011) and the Salzburg Statement on Shared Decision‐making. Charles *et al.* (1999) outlined a framework for SDM, which is schematically represented in Fig. 1. This four‐step model acknowledges that decision‐making is a fluid process for patients and describes three main approaches to decision‐making: the paternalistic approach, the shared approach and the informed approach. They discuss the concept of arriving at a ‘middle ground’ and indicate that SDM has an impact not only on clinical practice but also on the education of future medical practitioners and on research, including clinical trial patient accrual. In this review, all aspects of this framework will be analysed, specifically in the context of radiation therapy, which according to evidence‐based practice is one of a number of treatment options for the patient.

### 1.2 Radiation therapy in cancer management

Radiation therapy is recommended as part of treatment for more than 50% of cancer patients (Borras *et al.*, 2015a, 2015b,2015a, 2015b). However, one in four people who require radiation therapy does not receive it (Borras *et al.*, 2015a). While the reasons for this are multifactorial, including underinvestment in radiation therapy equipment and significant variations in access globally (Zubizarreta *et al.*, 2017), there is still an underutilisation of radiation therapy in so‐called ‘developed’ countries (Batumalai *et al.*, 2018; Borras *et al.*, 2015a), meaning that both patient‐ and physician‐related factors also have a role to play. In a Canadian study of the general public, Gillan *et al.* (2014) predominantly negative perceptions of radiation therapy were found leading the authors to conclude that radiation therapy has an international ‘image problem’. An Australian study (Sundaresan *et al.*, 2017) found that patients' decisions about whether or not to receive radiation therapy can be impacted by fear and anxiety about radiation therapy and perceived side effects. Others have reported that radiation oncology is a medical field that is largely unknown to the majority of patients (Shabason *et al.*, 2014). International advocacy campaigns such as *Targeting Cancer* in Australia and New Zealand (Cancer, 2019) and the European Society of Radiation and Oncology's (ESTRO) *Marie Curie Legacy Campaign* (2019) aiming to battle misinformation about radiation therapy in the public domain.

Exacerbating the issue is the fact that referral patterns to radiation oncology are largely based on the status of the referring institution as well as the knowledge of and perception of radiation therapy of the referring physicians (Morris *et al.*, 2018; Quek *et al.*, 2015). Using the Surveillance, Epidemiology and End Results (SEER) database of more than 85 000 physician visits of patients with newly diagnosed early‐stage prostate cancer, Jang *et al.* (2010) found that over half of these patients saw a urologist only and that 34% of these went on to have a radical prostatectomy (RP). Of the 44% who saw both a urologist and radiation oncologist, 83% of these patients went on to have radiation therapy. Sundaresan *et al.* (2015) found that health professionals believed that perception of radiation therapy, including management of acute side effects and their impact on daily commitments, fear and anxiety about radiation therapy, treatment‐related travel, relocation and need for accommodation and disruption to work and family life, is moderate to significant influences on the uptake of radiation therapy.

### 1.3 Decision aids in radiation oncology

Shared decision‐making can take place with or without the use of ‘decision aids’. The International Patient Decision Aids Standards collaboration (IPDAS) (Collaboration, 2010) defines patient decision aids as:

tools designed to help people participate in decision‐making about health care options. They provide information on the options and help patients clarify and communicate the personal value they associate with different features of the options. Patient decision aids do not advise people to choose one option over another, nor are they meant to replace practitioner consultation. Instead, they prepare patients to make informed, values‐based decisions with their practitioner.

A decision aid can be presented as a video, interactive digital media or a printed handbook. What is critical is the inclusion of a personalised discussion of treatment options with the patient in the context of their value and belief systems and their preferences, as well as the medical evidence on which to base the decision (Berman *et al.*, 2016). In order to be effective, decision aids must provide all treatment options as well as the probabilities of benefits and harms of each option, should allow patients to reflect on their own values and guide them towards a shared decision with their physician (Hoffman *et al.*, 2018). The criteria for a quality decision aid according to the IPDAS are given in Table 1. The update of the IPDAS collaborative background document in 2012 (Volk *et al.*, 2013) outlined the importance of providing the user with high‐level scientific evidence, where it exists, and presenting all treatment options in a clear and unbiased manner. The issue of understandability of the aids for patients of all literacy and health literacy levels is frequently disregarded, and the IPDAS stipulate that decision aids should be written at a (US) grade 8 equivalent level or less according to readability score [Simple Measure of Gobbledygook (SMOG) or Fry], with probabilities being presented not only through a text, but also through visual diagrams and numbers.

**Table 1:** The quality components for decision aids by the IPDAS.

Dimension	Explanation
Information	Information should be provided about options in sufficient detail in order to make a specific decision
Probabilities	Outcomes probabilities should be presented
Values	Values should be clarified and expressed
Decision guidance	Structured guidance should be provided to deliberate and communicate
Development	A systematic development process should be utilised
Evidence	Evidence used should be presented
Disclosure	Transparency and disclosure are required
Plain language	Plain language only should be used
Decision support tool evaluation	Knowledge and a match between values and chosen treatment option
Test	For decision support tools specifically for screening or investigations

### 1.4 Patient‐reported outcome measures

While a detailed analysis of patient‐reported outcome measures (PROMs) is beyond the scope of this review, the inclusion of PROMs in radiation oncology practice indirectly aids in the SDM process. The use of PROMs puts the patient perspective front and centre of the entire process (Selby and Velikova, 2018) and assists in changing the clinician's mindset towards the patient in their interactions. Using PROMs, patients have the opportunity to systematically describe and report side effects in their own terms. Clearly, these outcomes are more meaningful to the individual and a significant improvement from the subjectivity associated with side effect reporting by clinicians who attribute a somewhat arbitrary severity score to that side effect. Validated patient‐reported outcome (PRO) instruments have been recommended for inclusion in routine oncology practice (Basch *et al.*, 2012). In radiation oncology, the recent IMPORT LOW (CRUK/06/003) Phase III randomised controlled trial (Bhattacharya *et al.*, 2019) in low‐risk breast cancer used validated PRO instruments to ascertain that partial breast and whole‐breast low‐dose groups reported fewer adverse events than the control group, receiving the standard whole‐breast dose of 40 Gray in 15 fractions. Interestingly, this study also identified baseline predictors for the reporting of adverse events post‐treatment. These factors were younger age, larger breast size/surgical deficit, lymph node positivity and higher levels of anxiety/depression. This finding illustrates the potential value of studies exploring PROMs to identify the most suitable decision aid approach for specific patient cohorts. In fact, the use of PROMs has been cited as having the ability to transform clinical practice across a range of medical specialties (Black, 2013).

### 1.5 Literature screening

A systematic approach to screening was undertaken in this review using covidence systematic review software (Covidence, 2019). Relevant literature was searched using the databases PubMed, Embase, CINAHL, Web of Science and Medline. The search terms and Boolean operations for each database can be found in Appendix S1. The last search was run on 8 August 2019. The initial number of results was 2261, yielding 1740 publications after deduplication. Each paper was reviewed by two of the authors using covidence systematic review software (Covidence, 2019), according to predetermined inclusion and exclusion criteria. Conflicts in consensus were adjudicated upon by the lead author.

### 1.6 Inclusion criteria

As stated previously, overlap of terms related to patient decision‐making in the radiation oncology literature is commonplace; therefore, all of these concepts were included in the search. Publications where radiation therapy was at least one of the treatment options, together with a focus on any of the following, were included in this review: shared decision‐making; patient information; patient advocacy; patient collaboration; clinical trial accrual; patient empowerment; or patient involvement. All cancer sites were included, and publications were limited to the last 10 years. All study methodologies were included in this review.

### 1.7 Exclusion criteria

Publications that were in a language other than English were excluded, as were, publications that were available in abstract only or were reported as conference proceedings. Publications that discussed cancer treatment regimens that did not include radiation therapy were also excluded.

### 1.8 Studies included

**Fig. 2:**
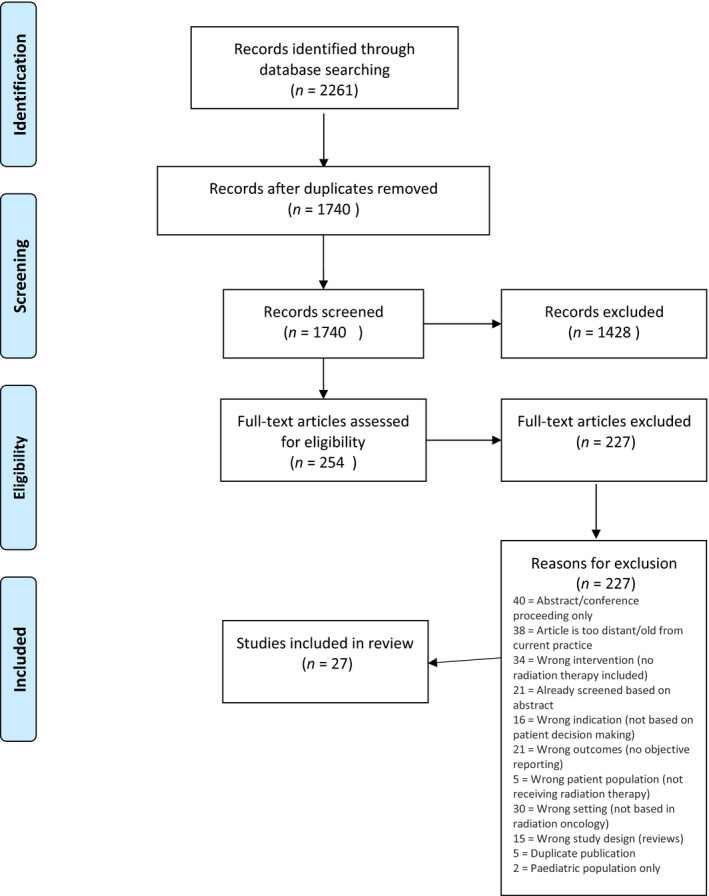
Flowchart of included studies. The studies included in the review and how they were selected are described.

A total of 1740 references were imported into the covidence systematic review software (Covidence, 2019) for screening. Five duplicates were removed. A total of 1731 studies were screened against title and abstract with 1428 of these excluded based on title and abstract screening. A total of 254 studies were assessed for full‐text eligibility. A total of 227 of these were excluded. Many studies excluded at this stage may have alluded to patient decision‐making, engagement or involvement in their title and abstract but full‐text eligibility screening indicated that radiation therapy was not an aspect of their care (*n* = 69). Other reasons for exclusion are given in Fig. 2. Twenty‐seven studies were deemed eligible for inclusion and are summarised in Table 2.

**Table 2:** Summary of included studies.

Author	Year	Article type	Cancer disease site	Main intervention reported	Main outcome reported	Impact on patient empowerment
Barry *et al*.	2010	Review/commentary	Prostate	DAs	With DAs, PSA screening interest declines	18 trials show DAs improve decision quality for PSA screening and are recommended by the American Cancer Society but are still not in routine use
Berry *et al*.	2011	Randomised control study	Localised prostate cancer	Internet‐based Personal Profile Prostate + usual education versus usual education alone	1. Decision‐making conflict score difference was borderline overall but significant for uncertainty (−13.3% variability from baseline) and values (−17.2% variability from baseline) subscales 2. Time to treatment was comparable between groups 3. Treatment choice – Brachytherapy chosen more often in the intervention group than the control group	Programme acceptability/usefulness was highly rated
Brom *et al*.	2014	Qualitative descriptive study	Metastatic colorectal cancer and glioblastoma multiforme (GBM)	Control of Preferences Scale	Preference for inclusion in treatment decisions in the palliative setting	Patients preferred their physicians to have a role in the decision‐making process. Own participation depended on how they saw their role or capabilities, depending on the phase of illness
Cuypers *et al*.	2016	Cross‐sectional study	Low and intermediate‐risk prostate cancer median 48 months post‐treatment	Control Preference Scale and EORTC QLQ‐INFO 25	Preference for inclusion in treatment decisions and evaluation of information received	Role preferences should be assessed and information tailored to patients to improve participation in treatment decision‐making
Cuypers *et al*.	2018	Cluster randomised control trial	Low‐ or intermediate‐risk prostate cancer patients with a minimum of two options for treatment	Usual care versus usual care and a DA about all treatments, values clarification exercises and a summary to bring to the next consultation	Decision regret and satisfaction with information received at time of treatment decision	No difference between reports of decision regret or information satisfaction between control and DA groups 12 months after treatment. Anxiety and depression at time of treatment decision can impact on decision regret and information dissatisfaction
Engebretson *et al*.	2016	Qualitative descriptive study	Pancreas	70 question survey by the Pancreatic Cancer Action Network	49.1% of respondents stated that they had never discussed clinical trials with a physician, despite NCCN guidelines on clinical trial participation in this disease site	Trial participation and potential improvement in outcomes for patients need to become part of the SDM process
Hermann *et al*.	2018	Cross‐sectional study	Any cancer site, though more than one third had breast cancer	Adapted version of the Control Preference Scale	33% of respondents were not involved in the decision‐making of their treatment to the extent that they wished	Not asking patients about their preferred involvement in cancer treatment decision‐making may lead to care that does not align with patients' wishes
Hopmans *et al*.	2015	Sequential mixed‐methods design using interviews and a survey	NSCLC stage I	Six qualitative themes covering SDM aspects were assessed	Four SDM‐related factors had sufficient internal consistency: 1. Guidance by clinician, 2. Conduct of clinician, 3. Preparation for treatment decision‐making, 4. Active role of patient in treatment decision‐making	Both surgical and radiation therapy treatment options were discussed with only 28.9% of respondents
Ihrig *et al*.	2010	Qualitative with semistructured interviews and a questionnaire	Localised prostate cancer	All aspects of the treatment decision process were assessed including the willingness to hypothetically enter a clinical trial	88% of respondents wishes to decide on their treatment option, together with their physician, however more than half of urologists recommended only a single treatment	Only 6% of patients would enter a hypothetical clinical trial, due to loss of control of randomisation
Keating *et al*.	2010	Population‐based cohort study	Lung and colorectal cancer	Modified version of the Control Preference Scale. Multinomial regression analysis to ascertain if characteristics of the decision influenced the patients' roles in the decision	10 939 treatment decisions of 5383 patients were analysed. Of these 38.9% were patient controlled, 43.6% were shared and 17.5% were physician controlled	Improved strategies for SDM are needed in situations where there is equipoise between treatments and in the event of terminal illness where cure is not possible
Kunneman *et al*.	2015	Audiotaping of physician‐patient consultations followed by patient survey	Rectal cancer	Number of value discussed was compared to age, gender, educational level and whether or not accompanied by a companion. No differences were observed	Patients' (*n* = 90) values were discussed in 32% of consultations, preferences in 12% or both in 10%. In 46% of consultations neither the patients' values or treatment preferences were addressed	Patients perceived a more active role in decision‐making when their values or preferences had been referred to during the consultation or when it was indicated that these would be considered in the decision‐making process
Kunneman *et al*.	2018	Qualitative. Patient Interviews followed by survey	Endometrial cancer	Treatment trade‐off method to assess the minimally desired benefit from vaginal vault brachytherapy in local control	44% of irradiated patients indicated that they felt they had no choice regarding vaginal vault brachytherapy. No significant differences between patients regardless of age, educational level, having a partner or children or comorbidity	Many irradiated patients felt they had lacked the space to think about and give their opinion on the benefits and harms of vaginal vault brachytherapy as well as participating in the treatment decision process to the extent that they would have wished
Lux *et al*.	2013	Qualitative questionnaire with hypothetical scenarios	Metastatic breast cancer	Patient questionnaire was distributed both online and in collaboration with patient representative groups	Patients have higher expectations from treatments in the metastatic breast cancer setting than do physicians. Younger age, age of children, participation in patient advocacy and educational level significantly impacted on expectations	Improvements are needed on both sides of the SDM process. Physicians need to be aware of the influence of personal aspects, such as age of children on the expectations of treatment outcome for metastatic breast cancer patients
Tong *et al*.	2016	Conjoint Preference Experiment of hypothetical lung cancer in current and former smokers	Early‐stage lung cancer	Conjoint analysis – a statistical technique using stated preferences and weighting their importance	Respondents were most likely to accept minimally invasive surgery, followed by SABR and lastly, thoracotomy	Even when the estimated complications and risk of locoregional recurrence were given to patients, 72% chose minimal surgery, 22.7% SABR and 5.3% thoracotomy
Kunneman *et al*.	2016	Audiotaping of physician and patient first consultations	Rectal and breast cancers	Observing Patient Involvement Scale (OPTION) to quantify to what extent physicians included patients in the decision‐making process	70% of consultations gave a reason for the visit. In only 3% of consultations was that a ‘treatment decision needed to be made’ In 44% of consultations, the reason given was to ‘explain the treatment details’. In 17%, it was stated that the reasons was that the patient was ‘there for treatment’ and no reason was given in 30% of consultations	In no consultation, including the 3% where a ‘decision needed to be made’ was the option of forgoing (neo)adjuvant treatment explicitly expressed
Lamers *et al*.	2016	Pragmatic cluster randomised control trial	Localised prostate cancer	Web‐based preference‐sensitive decision aid. Treatment preferences and patients' values were extracted from the DA. Urologists' treatment preferences were indicated at time of inclusion in the study	In 67% of 181 patients, treatment preference before DA use did not change after DA use	Agreement between the final treatment decision and the urologist's recommended treatment was lower than that between final treatment decision and preferred treatment after using the DA
Mokhles *et al*.	2018	Prospective observational cohort study	Early‐stage NSCLC	Questionnaire, control preference scale and decisional conflict scale	40% and 48% of patients receiving surgery and SABR respectively felt decisional conflict. 32% of patients receiving SABR felt unclear about personal values for benefits and side effects of treatment	Despite the majority of patients stating that they felt included in the decision‐making process, decision conflict was high in both surgical and SABR groups
O'Brien *et al*.	2013	Qualitative descriptive study	Early‐stage breast cancer with a moderate or high risk of recurrence	Semistructured interviews 2 weeks after the initial physician consultation	Women perceived themselves to be involved in treatment decision‐making both within the consultation process and outside of it	In this study, most women described the role of family and friends in making their decisions, known as ‘distributed decision‐making’
Pieterese *et al*.	2019	Adaptive conjoint analysis	Newly diagnosed rectal cancer patients	Stand‐alone online VCMs	Patients in the VCM group had significantly less decisional regret at 6 months than those in the control group	Half of the patients who completed the VCM found it difficult or distressing, indicating the difficulty for patients in making benefit/harm trade‐offs
Sattar *et al*.	2017	Qualitative study	Patients aged ≥ 65 years with breast, prostate, colorectal or lung cancer treated with wither curative or palliative intent	Semistructured qualitative interviews	Themes emerged in how older patients make treatment decisions. These were: ‘Trust in oncologist’, ‘Prolonging life’, ‘Expected outcomes of treatment’, ‘Other people's experience’, ‘Scepticism about going online’, ‘Assertion of independence’	The majority of older cancer patients were satisfied with their treatment decision process and voiced trust in their oncologists
Shabason *et al*.	2014	Cross‐sectional survey study	All cancer sites being treated with curative intent	3‐item scale to measure patient perception of radiation oncologist's SDM style Second questionnaire to evaluate perceived and desired control, satisfaction, fatigue, depression and anxiety	Of 305 participants, 31.3% reported experiencing SDM, 32.3% perceived control in treatment decisions and 76.2% were very satisfied with their radiation oncology care	A patient's perception of control in treatment decisions was associated with an increase in satisfaction, even when the patient did not express desire for control over decision‐making
Smith *et al*.	2017	Qualitative study with a phenomenological theoretical foundation	Prostate, breast, gynaecological, head and neck, colorectal and haematological malignancies and melanoma	Semistructured interviews	Most patients perceived the treatment decision as following the recommendation of the oncologist, with little discussion of benefit or harm or a sense that there was a decision to be made	Areas of uncertainty regarding radiation therapy were highlighted, including why it was recommended, how it works, the efficacy of treatment, the intensity of side effects and the risks of recurrence and radiation exposure
Sundaresan *et al*.	2017	RCT where patients received information on clinical trial participation with or without a decision aid (DA)	High‐risk prostate cancer patients postprostatectomy	Decision aid including questionnaire and reading material. Decisional conflict measured using the decisional conflict scale	Decisional conflict was significantly lower in the DA arm compared to control over 6 months	Proportion of patients recruited to the RCT was more than double in the decision aid arm (20.6%) compared to the control arm (9%)
van Stam *et al*.	2018	Control Preference Scale, decisional regret scale	Early‐stage prostate cancer	Questionnaires at baseline, 3, 6 and 12 months	87% of 454 patients reported being actively involved in treatment decision‐making	Active involvement was significantly associated with less decision conflict and decision regret
van Tol‐Geerdink *et al*.	2015	Multicentre trial on three sites with imbalanced randomisation (1 : 2)	Prostate cancer	Decision regret scale and three new scales focusing on process, option and outcome regret	The new regret scales help to distinguish separate aspects of regret	A trend towards lower option regret with a decision aid was observed in patients with serious morbidity
Veenstra *et al*.	2019	Population‐based survey study – part of the Individualised Cancer Care (iCanCare) Study	Breast cancer	Questionnaire with three domains of engagement in decision‐making developed from the concept of patient‐centred care	1203 patients and their key decision support persons (DSPs) were included. DSPs felt highly engaged (informed, involved and aware) in the decision‐making process although this varied with sociodemographic characteristics	Potential for interventions aimed specifically at DSPs to support patients in SDM
Wang *et al*.	2017	Qualitative survey	Breast cancer patients ≥ 65 years of age	Survey through interview, telephone or mail	More than 96% of respondents stated that they were the main decision‐makers in relation to having adjuvant radiation therapy or not	Older breast cancer patients may have more input into treatment decision‐making than previously anticipated

### 1.9 Aim and objectives

The aim of this review was to provide a narrative describing if, how and why patients contribute to decisions about their own care in radiation oncology. The specific objectives are to:

1. ascertain whether SDM occurs in radiation oncology practice and the role of decision aids in this process
2. describe the impact of SDM on cancer treatment selection
3. discuss whether SDM impacts on clinical trial patient accrual
4. identify gaps in scholarly evidence in relation to the field of radiation oncology and shared decision‐making.

## 2 Results

### 2.1 Decision aid paradigms

Several examples of the use of decision aids in radiation oncology SDM are reported in the literature with conflicting results. Berry *et al.* (2011) developed a Personal Patient Profile Prostate (P3P) online decisional aid, paired it with usual education procedures and compared its use in an experimental group to a control group receiving usual education only. Focusing specifically on radiation oncology endpoints, they illustrated an increase in the number of patients deciding to receive brachytherapy for their localised prostate cancer at a 6 months timepoint, relative to the control group.

It has been postulated that the use of decision aids might reduce decisional conflict and/or decision regret in patients following their treatment completion. Cuypers *et al.* (2019) in a longitudinal study of 384 prostate cancer patients, 111 who received usual care information and 273 who received usual care together with a decision aid at time of treatment decision found no difference in decisional regret nor dissatisfaction with information received between groups at 12 months of follow‐up. Men with higher baseline anxiety and depression at the time of the treatment decision did however report increased regret about their treatment decision and lower satisfaction with the information they had received. Lamers *et al.* (2017) report on the use of a Web‐based decision aid for deciding on the management of localised prostate cancer. The decision aid consisted of information about surgery, external beam radiation therapy (EBRT), brachytherapy and active surveillance as well as exercises for ‘values clarification’. Most men in this study selected their final treatment option after utilising the decision aid. In 67% of cases, the patient's choice did not change pre‐ and postcompletion of the decision aid. However, the correlation between the patient's final choice and the preference of the consulting urologist was lower after use of the decision aid, indicating that the decision aid could supersede the opinion of the urologist in the patients' final treatment choice. While many decision aids are made available online, the optimal format and delivery of decision aids are currently unclear in how they affect decision‐making processes as well as treatment selection (Barocas *et al.*, 2017).

Pieterse *et al.* (2019) developed an online stand‐alone values clarification method (VCM), outside of a decision aid, for newly diagnosed rectal cancer patients. At 6 months postconsultation, there was significantly less decisional regret in those who had completed the VCM versus those that had not. Half of those who had completed the VCM stated that they had found it distressing to do so, illustrating the impact that full consideration of benefits and harm of treatment can have on the psychological status of patients.

### 2.2 Role preference – passive, active or shared?

Cuypers *et al.* (2016) found that men with prostate cancer who stated they preferred a passive role in treatment decision‐making reported less overall satisfaction with the information they received at the time of decision‐making. These patients were older and less well educated than patients who stated that their preference was to actively take part in decision‐making. Patients had been diagnosed a median of 48 months previously. The authors postulated that the quality (rather than the quantity) of information that patients received was insufficient for aiding treatment decisions. Moreover, Charles *et al.* (1999) indicated that clinicians need to ascertain the role preference of patients in the decision‐making process at the time of treatment decisions. In an Australian survey of 416 cancer patients, one third reported dissatisfaction with the level of involvement that they had been presented with when making decisions regarding their treatment (Herrmann *et al.*, 2018). Age and gender had no significant impact on the observed discordance of patients with their role. In a large study of more than 5000 lung and colorectal cancer patients (Keating *et al.*, 2010) that examined patient feedback on almost 11 000 treatment decisions, most treatment decisions were found to have involved patients, being either patient controlled (38.9%), or shared (43.6%), with only 17.5% described as physician controlled. In the same study, patients who received radiation therapy as a treatment reported least patient control of the decision. This finding was also true for patients in the metastatic setting, where no treatment option would result in cure.

The decision to undergo short course pre‐operative radiation therapy in rectal cancer is a complex one for patients and physicians alike, as it is preference‐sensitive. While there is no evidence to support longer overall survival in those patients with rectal cancer who receive pre‐operative radiation therapy, there is evidence to support better local control with pre‐operative radiation therapy versus surgery alone though with an increased risk of sexual dysfunction and faecal incontinence (Kunneman *et al.*, 2015b). Kunneman *et al.* (2015a) report on 90 audiotaped consultations between radiation oncologists and patients in this scenario. Patients were subsequently followed up by survey to ascertain their satisfaction with their treatment decision. Oncologists and patients discussed patients' values in 32% of consultations, their treatment preferences in 12%, and both values and preferences in 10% of consultations. No discussion of patient values or treatment preferences occurred in 46% of recorded consultations. Educational status, gender, age or whether or not having a companion accompany the patient to the consultation did not seem to influence the discussion that occurred. Unsurprisingly, the follow‐up survey after treatment reported that those patients whose values and preferences had been addressed during the consultation perceived that they had had a more active role in their treatment decision.

A similar clinical scenario exists in the use of vaginal vault brachytherapy for high‐intermediate risk endometrial cancer patients. Brachytherapy is an internal delivery of radiation therapy using sources such as Iridium (Ir)‐192, implanted directly into or close to the tumour or area of risk of cancer spread. These sources produce gamma rays. While no evidence supports improved overall survival with the addition of vaginal vault brachytherapy for high‐intermediate risk endometrial cancer patients, radiation decreases local recurrence at the risk of increased toxicity due to mucosal atrophy. The treatment of a vaginal vault recurrence is however more intensive, requiring both EBRT and brachytherapy (Kunneman *et al.*, 2014). Of 95 patients who were interviewed and completed a questionnaire, 44% had received surgery only and 56% had received surgery and vaginal vault brachytherapy. Of the latter group, 42% stated that they had lacked time to think about the benefits and harms of treatment, 43% had not been afforded the opportunity to give their opinions on benefits and harms, and 45% did not have the opportunity to participate in SDM as much as they had wished (Kunneman *et al.*, 2014). Interestingly, Shabason *et al.* (2014) found in a cross‐sectional study of over 300 patients of all cancer types, patients who stated that they did not desire control over treatment decision‐making but received it reported more satisfied with their radiation oncology experience than those who did not perceive they had control. In fact, those who did not perceive control were likely to more often self‐report increased anxiety, depression and fatigue. These results were corroborated in the situation amongst early prostate cancer patients by van Stam *et al.* (2018) who found higher levels of decisional regret was significant amongst patients who did not perceive themselves to be actively involved in the decision‐making process, regardless of their stated decisional role preference for involvement. A Dutch study (van Tol‐Geerdink *et al.*, 2016) found a trend for use of a prior decision aid in specifically reducing instances of decision regret in a group of men who had experienced or were still experiencing significant treatment side effects.

Shared decision‐making is a complex process, particularly in oncology practice where there are many situations in which more than one management option for the patient's disease exists. Competing treatments, with similar outcomes in terms of overall survival, may have different risk/benefit profiles where trade‐offs may be necessary or have no proven superiority for one treatment over another (Samson *et al.*, 2016).

Clinicians cannot assume that all patients wish to or should be active participants in choosing their own treatment. This desire for a less active decisional role may especially be true in the palliative care setting, where treatment options can change and the number of options become fewer as the disease progresses. Brom *et al.* (2014) using the Control of Preferences Scale found that, as alluded to by the Charles SDM model (Charles *et al.*, 1999), decisions are fluid over time. This qualitative, descriptive study in metastatic colorectal and glioblastoma multiforme patients (both very poor prognostic groups) found that patients valued the input of their physician based on their medical expertise. With respect to their own part in decision‐making, this role varied depending on how they viewed their capacity for input at different timepoints through the disease experience. Lux *et al.* (2013) found a significant discrepancy between the expectations of patients and physicians in the management of metastatic breast cancer. Patients had much higher expectations of treatments to prolong life than did the treating physicians. Across all types of cancer treatments, 50% of patients expected an increase in overall survival of more than 12 months, and this expectation was only reported in 7–30% of physicians, indicating a failing in the SDM process. In a systematic review by Puts *et al.* (2010), most older adults with cancer accepted the treatment recommendation of their physician. However, in a study of 93 older breast cancer patients (Wang *et al.*, 2017), more than 96% indicated that they were the main decision‐maker as to whether they received adjuvant radiation therapy or not. The study by Sattar *et al.* (2018) illustrated some scepticism of older adults towards online resources, which should be considered when deciding on the best methodology for distribution of decision aids in this population.

A recent development in the field of radiation oncology is the positive response of stage I non‐small‐cell lung cancer (NSCLC) patients to the delivery of few, large doses of highly focused and targeted radiation called stereotactic ablative radiation therapy (SABR). Initially, this treatment option was specifically indicated for those who were medically inoperable or declined surgery. Now, some physicians believe that there is near equivalence between these two treatment options. However, Hopmans *et al.* (2015) reported that just under one third of 76 stage I NSCLC patients had had both treatment options discussed with them. In the same anatomic site, Tong *et al.* (2016) put hypothetical lung cancer treatment scenarios to current and former smokers and despite the benefits of SABR explicitly being explained to patients, more than 70% would still choose minimally invasive surgery as their primary treatment option. This opinion also applied to respondents who, due to self‐reported comorbidities, would most likely receive SABR for early lung cancer. To further complicate the issue, a Dutch study (Mokhles *et al.*, 2017) found that even when the majority of patients felt involved in the decision‐making process, 40% of 55 patients who received surgery for early‐stage NSCLC and 40% of 29 patients who received SABR reported decisional conflict following treatment. Decisional conflict was reported as being caused by uncertainty and being uninformed, illustrating that although their satisfaction with being actively involved in the decision process was high, these patients perceived that they received a lack of specific information, which may have contributed to the high levels of post‐treatment decisional conflict. It must be acknowledged that a cancer diagnosis is a time of significant uncertainty for all patients and some of this uncertainty cannot fully be eliminated during the treatment decision process.

### 2.3 Shared decision‐making and participation in clinical trials

Recruitment of patients to clinical trials in oncology is suboptimal and is cited as being as low as < 1% in the United States (Al‐Refaie *et al.*, 2011). The median actual accrual rate to Trans‐Tasman Radiation Oncology Group (TROG)‐sponsored trials from 2010 to 2012 has been cited as only half of the median expected accrual rate (Christie, 2013). Engebretson *et al.* (2016) surveyed US‐based pancreatic cancer patients and caregivers about their knowledge of clinical trials. Given its poor prognosis, current treatment guidelines recommend that patients with pancreatic cancer be enrolled in clinical trials (Tempero *et al.*, 2017). However in this survey of 184 patients and 213 caregivers, only 30% of diagnosing clinicians offered the patients any treatment options at diagnosis. Only 23.7% of patients reported having clinical trials discussed with them at the time of diagnosis or prior to the first treatment. Coupled with this, the loss of control due to randomisation cannot be underestimated in the inclusion of patients in clinical trials. When asked if they would enter a hypothetical clinical trial randomising to either RP or EBRT, only 6% of 31 men with localised prostate cancer (18 of whom went on to have RP and 13 EBRT) stated that they would consent to such a study (Ihrig *et al.*, 2009). Manne *et al.* (2015) also cite fear of side effects, worry about health insurance and efficacy concerns as significant barriers to patient accrual in oncology clinical trials.

It should be also be acknowledged that not all patients have access to clinical trial enrolment at all radiation oncology centres and that there is not always a suitable trial for every patient.

Decision aids as part of an SDM process may influence patient accrual in clinical trials. Sundaresan *et al.* (2011) found that the use of a decision aid in men with high‐risk prostate cancer following prostatectomy more than doubled the number of patients who consented to take part in the RAVES (Radiotherapy – Adjuvant versus Early Salvage) trial compared to those men who received standard trial information.

### 2.4 Shared decision‐making and treatment received

The first step in SDM is for the clinician to make the patient aware that the reason for the consultation is to arrive at a decision about treatment. A Dutch study (Kunneman *et al.*, 2016) involving a total of 100 patients that opted for neoadjuvant short‐course radiation therapy for rectal cancer, or adjuvant chemotherapy for breast cancer analysed audio‐recorded first consultations and reported that the option to forego treatment was not explicitly stated to any of the patients. Only 3% of consultations made clear that a treatment decision had to be made at the consultation, with 44% of consultations stating that their purpose was to ‘explain the treatment’.

A Canadian study (O'Brien *et al.*, 2013) found that the majority of 19 patients with early‐stage breast cancer who were interviewed about their perceptions of being involved in their treatment decision‐making had a positive opinion about their involvement. Interestingly, the authors refer to the fact that most patients do not make their treatment decisions in isolation, but rather with input from family and friends, a phenomenon known as ‘distributed decision‐making’. Veenstra *et al.* (2019) also explored the concept of a key ‘decision support person’. They found that having a highly informed decision support person resulted in higher odds of a patient making a deliberate decision.

Sattar *et al.* (2018) conducted a qualitative study looking at how older adults make cancer treatment decisions, and reported that trust in their oncologist was the dominant theme in decision‐making. Other themes included the prolongation of life, expected treatment outcomes, scepticism about online resources, the experiences of others and the assertion of independence. An Australian study (Smith *et al.*, 2017) found that patients who had undergone radiation therapy still had uncertainty over fundamental questions, including how treatment worked and the intensity of side effects. One initiative to counter this problem is the Australian ‘Radiation Therapist (RT) Prepare’ programme, which provides communication skills training for radiation therapists to prepare patients for treatment and to respond to emotional cues (Halkett *et al.*, 2018).

## 3 Discussion

### 3.1 Opportunities for shared decision‐making

The underutilisation of radiation therapy in developed countries may be attributable to both physician and patient‐related factors. The results of the study by Keating *et al.* (2010) where patients who received radiation therapy reported having the least patient control is an example of this. While not specified by the authors, it could be postulated that the perception and lack of knowledge of radiation therapy in the general community influenced some of the results of this study. Chemotherapy, which typically receives significant positive media coverage relative to radiation therapy, was shown to be associated with patients making far more self‐controlled decisions about their treatment compared to radiation therapy, in this study of lung and colorectal cancer patients.

Both radiation and medical oncologists have to accept responsibility for missed opportunities for SDM as demonstrated by Kunneman *et al.* (2016). Not one consultation recorded in this study explicitly included an explanation that patients had a choice between accepting or rejecting short‐course neoadjuvant radiation therapy for rectal cancer or, for breast cancer patients, that adjuvant chemotherapy had benefits and harms. As pointed out by the authors, not only is this a missed opportunity for SDM but it also puts the validity of informed consent into question. It is likely that treating clinicians had discussed the patients' cases in a multidisciplinary team meeting and therefore, in good faith, were presenting the patient with the outcomes of that meeting. However, the patient's opinion, values and concerns and ultimately their role in the decision‐making process were never brought into focus in the consultation, highlighting the point that where options exist, health professionals are not able to complete decision‐making on behalf of patients. Patient acceptability of side effects can change over time and what may not appear important at the time of treatment decision‐making can become extremely important post‐treatment; hence, the need for full and detailed information by the clinician on what accepting a treatment will mean in the future. Examples given in a recent review on cancer survivorship (Shapiro, 2018) include premature ageing and associated comorbidities postchemotherapy, increased incidence of cardiac events postchemotherapy and radiation therapy in thoracic patients, increase in sarcopaenia following chemotherapy and increase in distress following all cancer treatments. The cost, both personal and financial of such late outcomes for patients, cannot be underestimated, and therefore, full disclosure of the potential of such effects at time of treatment decision is necessary. Financial barriers cannot be underestimated and vary significantly by country. Similarly, the desire for an active role in decision‐making can also change over time as seen in breast cancer and this must also be considered (Hack *et al.*, 2006).

There are, however, reports of novel, patient‐focused, multidisciplinary and true SDM structures in place in oncology. Patrikidou *et al.* (2018) report on a combined urologist/radiation oncologist second opinion clinic in France, where patients with localised prostate cancer can choose to see a urologist and a radiation oncologist in a combined appointment. All patients have seen a urologist previously and are seeking a second opinion. A 2‐year evaluation of this service found that 55% of the 134 respondents surveyed had treatment options offered to them that had not been discussed at their initial consultation. Satisfaction with the service was extremely high at over 96%. Coupled with the input of other clinical specialists at time of consultation, this service may be a model of care to offer all patients and all treatment sites at initial consultation. Currently, this model is recommended in the National Institute of Clinical Excellence (NICE) prostate cancer guidelines (NICE, 2019).

### 3.2 Methods to improve the shared decision‐making process

Whether or not decision aids are helpful in SDM is ambiguous at present with some positive and some negative results reported. A systematic review and meta‐analysis of the value of decision aids in prostate cancer (Violette *et al.*, 2015) concluded that the key findings are the high risk of bias (related to concealment of allocation and blinding of outcome assessors), high variability in constructs measured and instruments used and the variability in study findings. Such methodological issues need to be addressed in future studies analysing the effect of decision aids on the SDM process before there is conclusive evidence to advocate for their routine use. In tandem with this uncertainty is the lack of experience and involvement with decision aids on the part of treating physicians as described by Wang *et al.* (2015), who surveyed radiation oncologists and urologists treating prostate cancer in the United States. Of 641 respondents, equally distributed between radiation oncologists and urologists, 35.5% stated that they currently used decision aids in their consultations with patients yet only 9.2% were confident that decision aids helped to improve treatment decisions. The authors attributed the low rate of use of decision aids to the lack of familiarity of such tools amongst the treating physicians.

Having a validated tool and correct delivery method for decision aids as well as a sound methodology to determine personalised treatment based on trade‐offs are all important for SDM in radiation oncology. So too is the provision of individualised information that is actually required by patients approaching a treatment decision. This ideal however is complex, as illustrated by Rüesch *et al.* (2014) in determining the information sought by early‐stage prostate cancer patients compared to what healthcare professionals (urologists, radiation oncologists, radiation therapists, nurses, medical oncologists and general practitioners) perceived to be important. This study found that the information needs of early‐stage prostate cancer patients are extremely heterogeneous and health professionals only weakly agreed on the topics of most importance for patients. Coupled with this was the finding that even within the same specialty, health professionals counsel patients in an inconsistent manner. Involvement of former patients in definition of decision aids therefore is paramount. A recently developed decision aid for advanced laryngeal carcinoma (Petersen *et al.*, 2019) shows promise in that it was specifically designed in collaboration with head and neck surgeons, radiation oncologists and former patients who had had total laryngectomy or chemoradiation therapy for their laryngeal cancer. During testing phases of the aid, the authors made considerable changes on accessibility of the information provided based specifically on the contribution of former patients. Less text and more animations were included to improve the comprehension of the information.

**Fig. 3:**
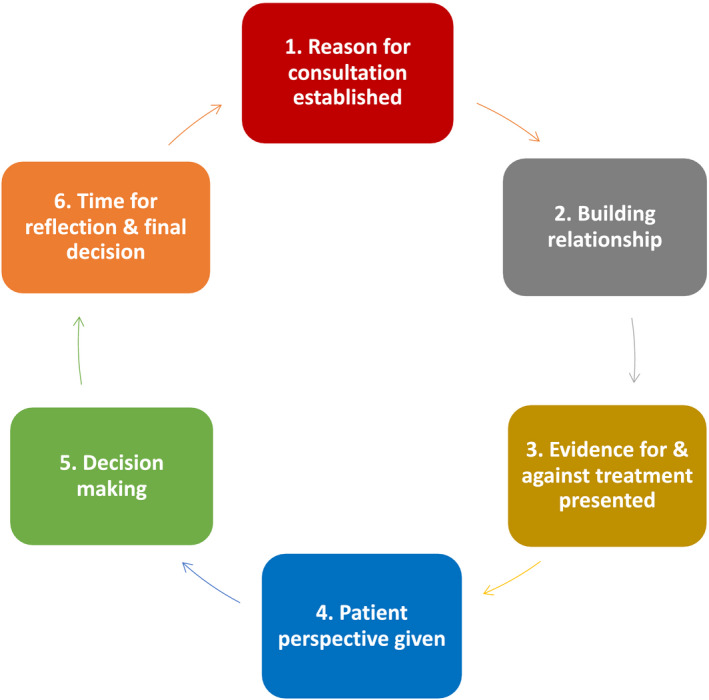
Shared decision‐making coding system (Singh *et al.*, 2010). The steps in this SDM model are outlined, starting at stating the reason for the patient–clinician interaction, and working through to reflection on the decision the patient has taken.

One method to change SDM practice is the use of a specific SDM coding system. Singh *et al.* (2010) defined an oncology‐specific coding framework, including six main areas, as given in Fig. 3. First, the reason for the consultation should be established; second, the clinician–patient relationship is built; third, the evidence is presented for and against treatment; fourth, the physician gains the patient's perspective. The last two steps involve making the actual decision including discussion of side effects and patient values and finally, a discussion of timing allowing the patient some time until the next visit before making a decision, if s/he so chooses. Such a framework ensures that all aspects of the SDM process are included and minimises the potential for the clinician to overlook any area. Another option described by Pieterse *et al.* (2010) is that of ‘adaptive conjoint analysis’, a technique that elicits preferences involving trade‐offs between different aspects of the treatment decision and preferences reported at the outset appear robust as time progresses. It can capture individual preferences independent of treatment experience in former patients.

On a wider scale, Chiew *et al.* (2018) integrated conceptual framework for quality in cancer care, with 12 domains including patient experience and satisfaction, appropriateness of care and guideline adherence, multidisciplinary and co‐ordinated care and patient‐centred outcomes is useful for the definition of quality metrics in cancer treatment and care.

The results of studies of van Stam *et al.* (2018) and Shabason *et al.* (2014) indicate that patients should be encouraged to take some part in the decision‐making process, regardless of their stated preference because in these studies, involvement leads to fewer instances of decisional conflict and regret and to increased satisfaction with treatment.

One barrier to improving the empowerment of patients in radiation oncology that is not discussed in the literature is the pressured and emotional environment in which most radiation oncology clinicians work. Clinicians who have to cope with the death of patients (Granek *et al.*, 2016), have extremely heavy workloads (Poulsen *et al.*, 2014) and symptoms of depression and burnout (Guerra and Patricio, 2019; Lazarescu *et al.*, 2018) lead to environments that are stressful for clinicians and patients alike. In turn, this setting is likely not conducive to providing patients with opportunities to share their perspectives and values, to fully consider the information provided to them or to reflect upon potential decisions.

## 4 Conclusion

This review dealing with empowering patients around decision‐making in radiation oncology reveals that the current status of SDM within the discipline is ad hoc at best. The literature indicates that the incidence of both decision regret and decisional conflict lessens when patients are involved in decisions about their own cancer treatment, regardless of whether they specifically choose to have an active role or not in the decision‐making process. The benefit of decision aids in radiation oncology is currently ambiguous. Few decision aid studies reported consider the literacy level of the patient who will use them. Ensuring comprehensibility of decision aids for all patients across various literacy and health literacy levels should be a focus point of future effort in this field.

Measures to address suboptimal SDM in radiation oncology might include: providing expert clinical support staff, for instance specialist nurses or radiation therapists, who can fully discuss all treatment options; development, testing and training around the use of effective tailored decision aids for patients, education of clinicians around the value and methods of SDM and provision of suitable environments and follow‐up for patients and their decision support persons to allow their effective participation in SDM. Increasing awareness of radiation therapy amongst the general public through international campaigns such as Targeting Cancer (www.targetingcancer.com.au) and the Marie Curie Cancer Campaign (www.mariecurielegacy.org) will bring focus to this key treatment modality and further help to empower future patients to consider the potential of radiation therapy as part of their cancer treatment.

Finally, the authors make the following recommendations to improve the current status of SDM for patients due to receive or potentially suitable for radiation therapy:

1. National Cancer Plans need to include specific focus on the important topic of SDM in order that appropriate investment is made into the necessary research and implementation of evidence‐based strategies. Specific attention placed on decision‐making involving radiation therapy as an alternative or adjuvant therapy to surgical and systemic cancer treatments will be key.
2. An agreed taxonomy is developed around the related but distinct concepts of patient empowerment, engagement and other terms closely linked to SDM. A sound qualitative method such as a Delphi consensus process including expert, clinician and patient input might be suitable and would improve standardisation of further research and reporting in this area. Such a classification may have value outside the field of radiation oncology as well.

## Conflict of interest

The authors declare no conflict of interest.

## Supporting information


**Appendix S1.** Search terms used per database.
