# Supplementary material for: Empowering patients in decision‐making in radiation oncology – can we do better?
Source: Mol Oncol. 2020 Apr 13;14(7):1442–60. doi: 10.1002/1878-0261.12675 (PMC7332211; doi:10.1002/1878-0261.12675)
Supplement: Supplementary file 1 — Appendix S1. Search terms used per database. [file MOL2-14-1442-s001.docx]

**Appendix 1**

**Search terms used per database.**

**EMBASE**

1. 'radiotherapy'/exp

2. (‘Radiation therapy’ OR radiotherapy OR ‘radiation oncology’):ti,ab

3. #1 OR #2

4. 'patient decision making'/exp OR 'shared decision making'/exp OR 'decision making'/exp

5. (joint-decision* OR shared-decision* OR ‘patient* decision*’):ti,ab

6. #4 OR #5

7. 'patient participation'/exp OR 'empowerment'/exp OR 'patient preference'/exp OR 'patient satisfaction'/exp OR 'patient information'/exp OR 'patient right'/exp OR 'patient-reported outcome'/exp OR 'patient advocacy'/exp OR 'patient education'/exp

8. (Patient* NEAR/2 (particpat* OR empower* OR engage* OR decision* OR control OR right*)):ti,ab

9. (‘Patient involvement’ OR ‘patient information’):ti,ab

10. (Patient* NEAR/3 (educat* OR leaflet* OR brochure* OR advocacy OR knowledge OR decision* OR preference OR decision-aid*)):ti,ab

11. #7 OR #8 OR #9 OR #10

12. #3 AND #6 AND #11

13. 'treatment planning'/exp OR 'radiotherapy planning system'/exp

14. ((Treatment OR therapy OR radiotherapy OR ‘radiation therapy’) NEAR/2 plan*):ti,ab

15. #13 OR #14

16. #12 AND #15

**OVID Medline**

1. exp Radiotherapy/

2. (Radiation therapy OR radiotherapy OR radiation oncology).ti,ab.

3. or/1-2

4. Decision Making/

5. (joint-decision* OR shared-decision* OR patient* decision*).ti,ab.

6. or/4-5

7. Patient Participation/ OR "patient acceptance of health care"/ or exp patient satisfaction/ OR Patient Education as Topic/ OR Patient Rights/ OR patient reported outcome measures/ OR Patient Advocacy/ OR exp Patient-Centered Care/

8. (Patient* adj2 (particpat* OR empower* OR engage* OR decision* OR control OR right*)).ti,ab.

9. (Patient involvement OR patient information).ti,ab.

10. (Patient* adj3 (educat* OR leaflet* OR brochure* OR advocacy OR knowledge OR decision* OR preference OR decision-aid*)).ti,ab.

11. or/7-10

12. and/3,6,11

**Web of Science**

TS= ((“Radiation therapy” OR radiotherapy OR “radiation oncology”) AND (joint-decision* OR shared-decision* OR “patient* decision*”) AND ((Patient* NEAR/2 (particpat* OR empower* OR engage* OR decision* OR control OR right*)) OR (“Patient involvement” OR “patient information”) OR (Patient* NEAR/3 (educat* OR leaflet* OR brochure* OR advocacy OR knowledge OR decision* OR preference OR decision-aid*))))

**CINAHL**

1. (MH "Radiotherapy+") OR (MH "Radiation Oncology")

2. TI (“Radiation therapy” OR radiotherapy OR “radiation oncology”) OR AB (“Radiation therapy” OR radiotherapy OR “radiation oncology”)

3. S1 OR S2

4. (MH "Decision Making, Patient") OR (MH "Decision Making, Shared") OR (MH "Decision Making") OR (MH "Decision Support Techniques")

5. TI (joint-decision* OR shared-decision* OR “patient* decision*”) OR AB (joint-decision* OR shared-decision* OR “patient* decision*”)

6. S4 OR S5

7. (MH "Consumer Participation") OR (MH "Empowerment") OR (MH "Patient Satisfaction+") OR (MH "Patient Education") OR (MH "Patient Rights") OR (MH "Patient Autonomy") OR (MH "Patient-Reported Outcomes") OR (MH "Patient Advocacy")

8. TI (Patient* N2 (particpat* OR empower* OR engage* OR decision* OR control OR right*)) OR AB (Patient* N2 (particpat* OR empower* OR engage* OR decision* OR control OR right*))

9. TI (“Patient involvement” OR “patient information”) OR AB (“Patient involvement” OR “patient information”)

10. TI (Patient* N3 (educat* OR leaflet* OR brochure* OR advocacy OR knowledge OR decision* OR preference OR decision-aid*)) OR AB (Patient* N3 (educat* OR leaflet* OR brochure* OR advocacy OR knowledge OR decision* OR preference OR decision-aid*))

11. S7 OR S8 OR S9 OR S10

12. S3 AND S6 AND S11
